# Pelagic organisms avoid white, blue, and red artificial light from scientific instruments

**DOI:** 10.1038/s41598-021-94355-6

**Published:** 2021-07-22

**Authors:** Maxime Geoffroy, Tom Langbehn, Pierre Priou, Øystein Varpe, Geir Johnsen, Arnault Le Bris, Jonathan A. D. Fisher, Malin Daase, David McKee, Jonathan Cohen, Jørgen Berge

**Affiliations:** 1 Centre for Fisheries Ecosystems Research, Fisheries and Marine Institute of Memorial University of Newfoundland, St. John’s, NL Canada; 2 Department of Arctic and Marine Biology, UiT the Arctic University of Norway, Tromsø, Norway; 3 Department of Biological Sciences, University of Bergen, Bergen, Norway; 4 Norwegian Institute for Nature Research, Bergen, Norway; 5 Centre for Autonomous Marine Operations and Systems, Department of Biology, Norwegian University of Science and Technology, Trondheim, Norway; 6 University Centre in Svalbard, Longyearbyen, Norway; 7 Physics Department, University of Strathclyde, Glasgow, Scotland, UK; 8 School of Marine Science and Policy, University of Delaware, Lewes, USA

**Keywords:** Behavioural ecology, Marine biology

## Abstract

In situ observations of pelagic fish and zooplankton with optical instruments usually rely on external light sources. However, artificial light may attract or repulse marine organisms, which results in biased measurements. It is often assumed that most pelagic organisms do not perceive the red part of the visible spectrum and that red light can be used for underwater optical measurements of biological processes. Using hull-mounted echosounders above an acoustic probe or a baited video camera, each equipped with light sources of different colours (white, blue and red), we demonstrate that pelagic organisms in Arctic and temperate regions strongly avoid artificial light, including visible red light (575–700 nm), from instruments lowered in the water column. The density of organisms decreased by up to 99% when exposed to artificial light and the distance of avoidance varied from 23 to 94 m from the light source, depending on colours, irradiance levels and, possibly, species communities. We conclude that observations from optical and acoustic instruments, including baited cameras, using light sources with broad spectral composition in the 400–700 nm wavelengths do not capture the real state of the ecosystem and that they cannot be used alone for reliable abundance estimates or behavioural studies.

## Introduction

In marine environments, artificial light drastically impacts the behaviour of both pelagic^1^ and benthic organisms^2^, but behavioural responses to artificial light vary among taxa. For instance, copepods^3^, Atlantic cod *Gadus morhua*^4^, and seabream *Sparus auratus*^5^ avoid light sources while herring *Clupea harengus*^6^, krill^4^, snow crab *Chionoecetes opilio*^7^, and grey mullet *Mugil cephalus*^5^ are attracted. Efficiency of fishing gears targeting the latter can thus be improved by including light beams or strobes^8^ or by including bioluminescent light^9^. It has also been suggested to use the light avoidance behaviour of certain species to herd them inside nets^10,11^ or classify their acoustic signal^12^. In addition to interspecific differences in responses to light^13^, intraspecific variation could also complicate interpretations of responses to artificial light.

Despite a growing body of literature reporting behavioural disturbance of marine organisms exposed to artificial light, external light sources remain widely used in oceanography and marine ecology studies. Recent advances in optical technology, combined with the increased desire to use non-lethal observation approaches, have driven the development of new sensors and instruments to document marine ecosystems^14^, but these instruments generally require an external light source. Optical probes such as the Underwater Vision Profiler^15^, the Laser-Optical Plankton Counter^16,17^, the Video Plankton Recorder^18^, and the Light frame On-sight Key species Investigation system^19,20^ all use light sources and optical sensors to assess the vertical distribution and abundance of zooplankton in the water column and use artificial light to discern the silhouette of animals. Researchers and the industry alike increasingly use High Definition (HD) video cameras or stereo-cameras mounted on trawls to document the catchability of different species or size classes of fish^11,21,22^. Baited cameras, drop-camera and remotely operated vehicles also rely on HD video cameras to assess the occurrence, behaviour and abundance of fish^23–26^. Most camera systems rely on external light sources to distinguish and identify marine animals at depth. Although previous studies have raised concerns about the impact of artificial visible light on measurements from optical instruments^22,27–30^, these biases have rarely been quantified^14^. Nonetheless, artificial lighting is assumed to be the main source of biases in fish surveys using cameras and underwater vehicles^13,31,32^.

The use of red light has been suggested for marine surveys requiring external light sources because it is assumed that most species do not react as much to red light as to shorter wavelengths, such as blue or green^22,28,32^. In support of this hypothesis, Peña et al*.*^12^ and Underwood et al.^11^ deployed oceanographic probes equipped with different light colours and showed that mesopelagic (200–1000 m) fish avoid white, blue and green, but not red light. Recent studies conducted in Svalbard (> 77°N) in January, during the polar night, revealed that the vast majority of the epipelagic (0–100 m) community exhibits a strong avoidance response when exposed to white artificial light from a research vessel^3^ and that this impact persists down to at least 200 m in open water^1^. Yet, behavioural responses to in situ light sources of different colours and irradiance levels on instruments lowered in the upper pelagic layers (< 200 m) remain poorly documented.

In January 2020, we conducted in situ experiments to study the behavioural responses of pelagic fish and zooplankton to different light colours in Svalbard (European high Arctic; 78–80°N). An acoustic probe equipped with external light sources was deployed in sound scattering layers and changes in the distribution and abundance of organisms were monitored using both the probe and a hull-mounted echosounder. Because of the continuous darkness and the absence of light pollution, the Arctic polar night represents an ideal environment to test the impact of artificial light on marine organisms^1^. However, it is possible that animals become acclimatized to the very low light levels prevailing during the polar night, which could increase their sensitivity to artificial light. To verify if our observations are also valid in other ecosystems with a day-night cycle, we conducted an additional experiment with a pelagic baited video camera equipped with white or red lights in coastal Newfoundland, Canada (48°N).

## Materials and methods

### Survey design

**Figure 1:**
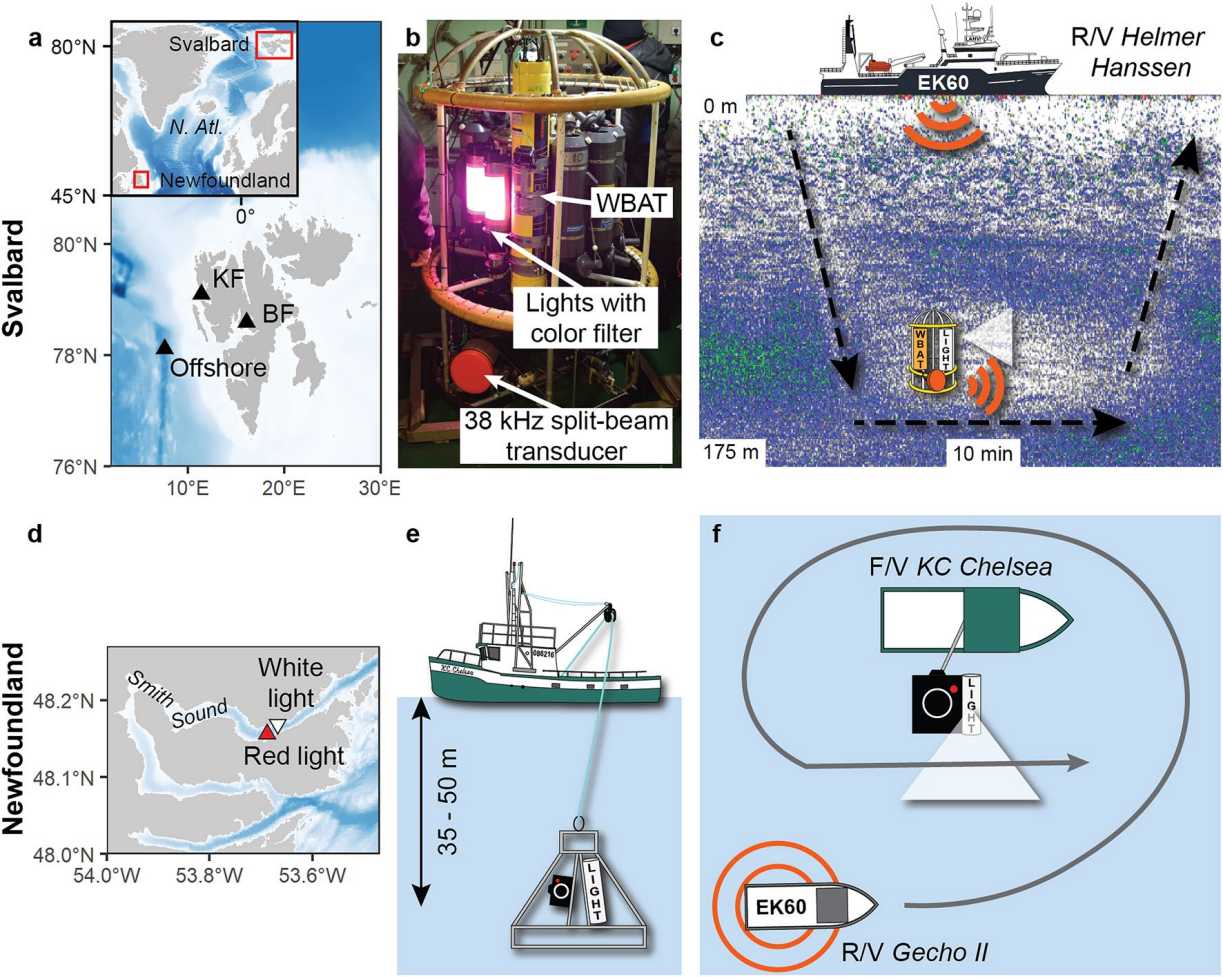
Map of the study area in Svalbard (**a**) and picture (**b**) and schematic (**c**) of the acoustic probe deployments. Map of the Newfoundland survey area (**d**) with side view (**e**) and top view (**f**) schematics of the deployments. KF- Kongsfjorden and BF- Billefjorden.

The Svalbard survey was conducted from the R/V *Helmer Hanssen* at three locations in western Svalbard between 9 and 14 January 2020 (Fig. 1a). The first two stations were located in Billefjorden and Kongsfjorden, two well-studied Arctic fjords with high abundances of fish and zooplankton^33–36^. The third station was located offshore (ca. 1000 m bottom depth), where the density of pelagic organisms was lower. At each station, we deployed an acoustic probe composed of a Wideband Acoustic Transceiver (WBAT; Kongsberg Maritime AS) mounted on a CTD-rosette and connected to a sideward-looking 38 kHz split beam transducer operated in broadband mode (Model ES38-18DK split-beam wideband (35–45 kHz); see Supplementary Table S1 online for details of the settings). Two custom-made LED lights normally used on camera systems mounted on trawls were installed on the CTD-rosette, close to the transducer and continuously illuminating the same direction when turned on (Fig. 1b). We alternated between no light, white, blue, and red external plastic film filters to obtain a diffuse light field with a given spectral composition, and in turn to measure the reaction of organisms to different wavelengths at each location. Because both the ship and the organisms drifted, we assumed that a different community was detected during each experiment (i.e. with each wavelength) and treated each experiment as independent. This assumption was supported by the recovery in backscatter between experiments. In Kongsforden and the offshore station, we used single or double layers of red filters to obtain different irradiance levels. No other lights were used on the CTD-rosette.

In addition to monitoring the behaviour of the organisms with the WBAT mounted on the rosette (i.e., acoustic probe), we recorded the change in volume backscattering strength (S_v_ in dB re 1 m^−1^) and distribution using an EK60 hull-mounted echosounder (Kongsberg Maritime AS, Norway) operating at 18 kHz and 120 kHz (Fig. 1c; see Supplementary Table S1 online for details of the settings). Note that the 38 kHz channel of the EK60 was turned off to avoid interference with the WBAT. In Billefjorden and Kongsfjorden, the experiment was repeated with the ship's lights turned on and off. At the offshore station, the experiment was conducted with the ship's lights turned off. The depth at which the probe was lowered (130 m in Billefjorden, 140 m in Kongsfjorden, and 125–130 m in the offshore area) was selected based on the depth of the sound scattering layer as seen on the hull-mounted echosounder. For each deployment, the probe remained at the same depth for 10 min while the ship was idle (Fig. 1c).

The Newfoundland survey was conducted in Smith Sound, a deep inlet with a maximum depth of 220 m, on November 20^th^ and 22^nd^, 2019 (Fig. 1d). A battery-operated HD video camera (Rayfin HDE-60, 70° diagonal field of view; SubC Imaging, Clarenville, NL, Canada) with frozen herring bait in its focal point was continuously illuminated with a SubC Imaging Aquorea white (5000 K temperature, 80° beam angle) LED external light on an aluminum frame^26^. The frame was then suspended between 35 and 50 m in the upper pelagic layer over the side of the F/V *KC Chelsea* (Fig. 1e). As a control, the R/V *Gecho II* passed three times close (within 2 m) to the *KC Chelsea* with the *Gecho II* hull-mounted EK60 echosounder operated at 38 kHz and 120 kHz (Supplementary Table S1 online) before deploying the camera. The camera was then deployed with white lights on (20 November) and then with a red plastic filter over the white light (22 November). For each experiment (white and red lights), we turned off the lights of both vessels and waited ca. 30 min between the camera deployment and the subsequent acoustic measurements to let the turbulence from the instrument dissipate. The RV *Gecho II* then passed another three times over the baited camera to record the change in backscatter with the EK60 echosounder (Fig. 1f). The experiments were conducted after sunset, around midnight UTC. All videos of the baited camera were observed frame by frame and scrutinized for pelagic organisms. Once the camera stabilized at the sampling depth, we tracked each organism entering the field of view to avoid double counting individuals, and identified animals to the lowest possible taxonomic level.

### Measurements of spectra and irradiance

For both the Svalbard and Newfoundland surveys, we measured the spectrum and irradiance of the external light source in the air and at 1 m distance for each colour using a SpectraPen LM500-UVIS spectroradiometer (Photon Systems Instruments, Czech Rep). The spectroradiometer was fitted with a cosine light corrector (180° viewing angle) providing spectral irradiance, E(λ) from 400–700 nm (full width at half maximum bandwidth of 7 nm) in energy (μW cm^−2^ nm^−1^) or quanta (μmol photons m^−2^ s^−1^ nm^−1^) mode. The irradiance integrated over the wavelength interval between 400–700 nm (visible light, Photosynthetically Active Radiation) is denoted E_PAR_ (μW cm^−2^).

### Measurements of optical properties of seawater

In Svalbard, absorption a(λ) and light backscatter b_b_(λ) profiles were recorded at 9 wavebands across the visible spectrum using an AC-9 spectrophotometer and a BB9 backscattering sensor, respectively (both Sea-Bird Scientific, USA). Data from both instruments were corrected for light absorption and scattering artefacts following standard manufacturer’s correction methods. The AC-9 was calibrated using freshly drawn Milli-Q ultrapure water of the ship. Temperature and salinity corrections were applied using concurrent data from Seabird SBE19Plus CTD profiles. The horizontal diffuse attenuation coefficient K_h_(λ)^37^, developed for extended parallel light beams, was estimated from a(λ) and b_b_(λ) using Eq. (1)^38^.

1 $$K_{h} (\lambda ) = \frac{{g[a(\lambda ) + b_{b} (\lambda )]}}{{\mu_{d} }}$$

Here, the parameter g = 1.0395 and μ_d_ is the mean cosine for downwards irradiance. We are considering light emitted horizontally from the CTD frame, but assume that this is equivalent to having the light source at zenith (θ_sw_ = 90°) and μ_d_ is then obtainned from Eq. (2)^39^.

2 $$\mu_{d} = 0.827{\text{cos}}\theta_{sw} + 0.144$$

The horizontal spectral penetration distance, τ_h_(λ), is given by Eq. (3).

3 $$\tau_{h} (\lambda ) = \frac{1}{{K_{h} (\lambda )}}$$

and represents the path length over which irradiance drops to 1/e (~ 37%) of its initial value. It was not possible to directly validate the model of diffuse light attenuation and it is possible that it does not fully resolve all features of the system, for example beam spread from the light source. Nonetheless, we believe it provides reasonable estimates of horizontal light penetration in Svalbard waters. Unfortunately, the optical properties of seawater were not measured in Newfoundland.

### Acoustic analyses

The EK60 echosounders were calibrated prior to the surveys using the standard sphere method^40^. The WBAT was not calibrated and its backscatter data should be considered as relative rather than absolute values. Temperature-salinity profiles recorded with a Seabird 911 Plus CTD (Svalbard) and an RBR Concerto (Newfoundland) were used to calculate sound speed^41^ and the coefficient of absorption at each frequency^42^ used in calculations for both the EK60 and WBAT data.

All acoustic data were scrutinized and cleaned with Echoview 11. The avoidance behaviour from the acoustic probe was calculated by measuring the average range between the transducer of the WBAT and the first appearance of echoes at a − 90 dB Sv threshold in Billefjorden and Newfoundland, − 95 dB Sv threshold in Kongsfjorden, and a − 110 dB Sv threshold offshore. The S_v_ threshold was set to 10% of the average undisturbed backscatter at each location (i.e., we calculated the distance of avoidance based on a 90% reduction in backscatter). Calculations were conducted on the nominal frequency (38 kHz) of the pulse compressed wideband pings with a 1 s resolution. The range to the − 90 dB, − 95 dB or − 110 dB scattering threshold was extracted using Echoview's Best Bottom Candidate Line Pick algorithm with the settings listed in Supplementary Table S2 (online) before being smoothed over 15 pings.

We used Echoview’s algorithms to remove background noise with a minimum signal-to-noise ratio threshold of 10 dB^43^, and impulse noise^44^ in the EK60 data. The change in acoustic density of pelagic organisms exposed to different light colours and irradiance was measured by comparing the area backscattering coefficient (s_a_ in m^2^ m^−2^) of the EK60 when instruments with light were deployed with the s_a_ over 10 min before the experiments. The s_a_ was integrated over the depth of the sound scattering layer, i.e., between 60 m and the bottom (ca. 170 m) in Billefjorden, 70 m and 200 m in Kongsfjorden, 70 m and 180 m offshore, and 7 m and 80 m in Newfoundland.

### Statistical analyses

A first generalized linear mixed effects model was used to test for the effect of ship light (on/off) and probe light colours (no light, blue, white, red high irradiance, red low irradiance), and site (Billefjorden and Kongsfjorden) on the avoidance distance (*AD*). Because experiments with both ship light and probe light treatments were conducted only at Billefjorden and Kongsfjorden, we only included these two sites in the first model. The model was developed as follows:

$$AD = a_{ij} + \beta_{1} B_{ij} + \beta_{2} P_{ij} + \beta_{3} S_{ij} + ID_{j} + \varepsilon_{ijs}$$

where $\beta_{1} , \;\beta_{2} ,\;\beta_{3}$ are fixed effects coefficients for variables ship light (B), probe light colours (P), and sites (S), for observation *i* in experiment trial *j*. A random effect *ID*_*j*_ was added to control for the non-independency of observations within each experimental trial *j*. *ID*_*j*_ was assumed to be normally distributed with mean 0 and variance $\sigma^{2}$. The error term $\varepsilon_{ijs}$ was modeled using a Gamma distribution with a log link function. The error distribution was selected based on the inspection of model residuals.

Results from model 1 revealed that the ship light did not affect avoidance distance (Table 1). Unlike the ship light effect, probe light experiments were conducted across the three sites; therefore, a second model was run without the ship light covariate to include data from the three Svalbard sites (i.e.*,* Billefjorden, Kongsfjorden, and offshore). Generalized linear mixed effects models were computed using glmer function in the R Core team (2013) package lme4^45^. The significance of fixed effects was tested with the Wald-Z test^46^.

**Table 1:** Fixed-effect coefficient estimates of the generalized linear mixed-models of light avoidance. *BF *Billefjorden, *KF* Kongsfjorden, and *OS* Offshore.

Model 1:	Reference is ship light = off, Probe light = off, Site = BF					Model 2:	Reference is Probe light = off, Site = BF				
Covariate	Level	Estimate	SE	z value	Pr ( >|z|)	Covariate	Level	Estimate	SE	z value	Pr ( >|z|)
Intercept		3.16	0.09	34.93	< 0.0001	Intercept		3.13	0.1	30.16	< 0.0001
Ship light	On	0.05	0.06	0.81	0.42	Probe light	Blue	0.82	0.13	6.33	< 0.0001
Probe light	Blue	0.74	0.11	6.72	< 0.0001		White	0.75	0.13	5.88	< 0.0001
	White	0.71	0.11	6.76	< 0.0001		Red high	0.71	0.13	5.43	< 0.0001
	Red high	0.62	0.11	5.54	< 0.0001		Red low	0.36	0.16	2.25	0.025
	Red low	0.07	0.15	0.44	0.659	Site	KF	0.35	0.09	3.66	< 0.0001
Site	KF	0.43	0.07	5.88	< 0.0001		OS	− 0.47	0.10	− 4.57	< 0.0001

## Results

### Light climate and optical properties of seawater

**Figure 2:**
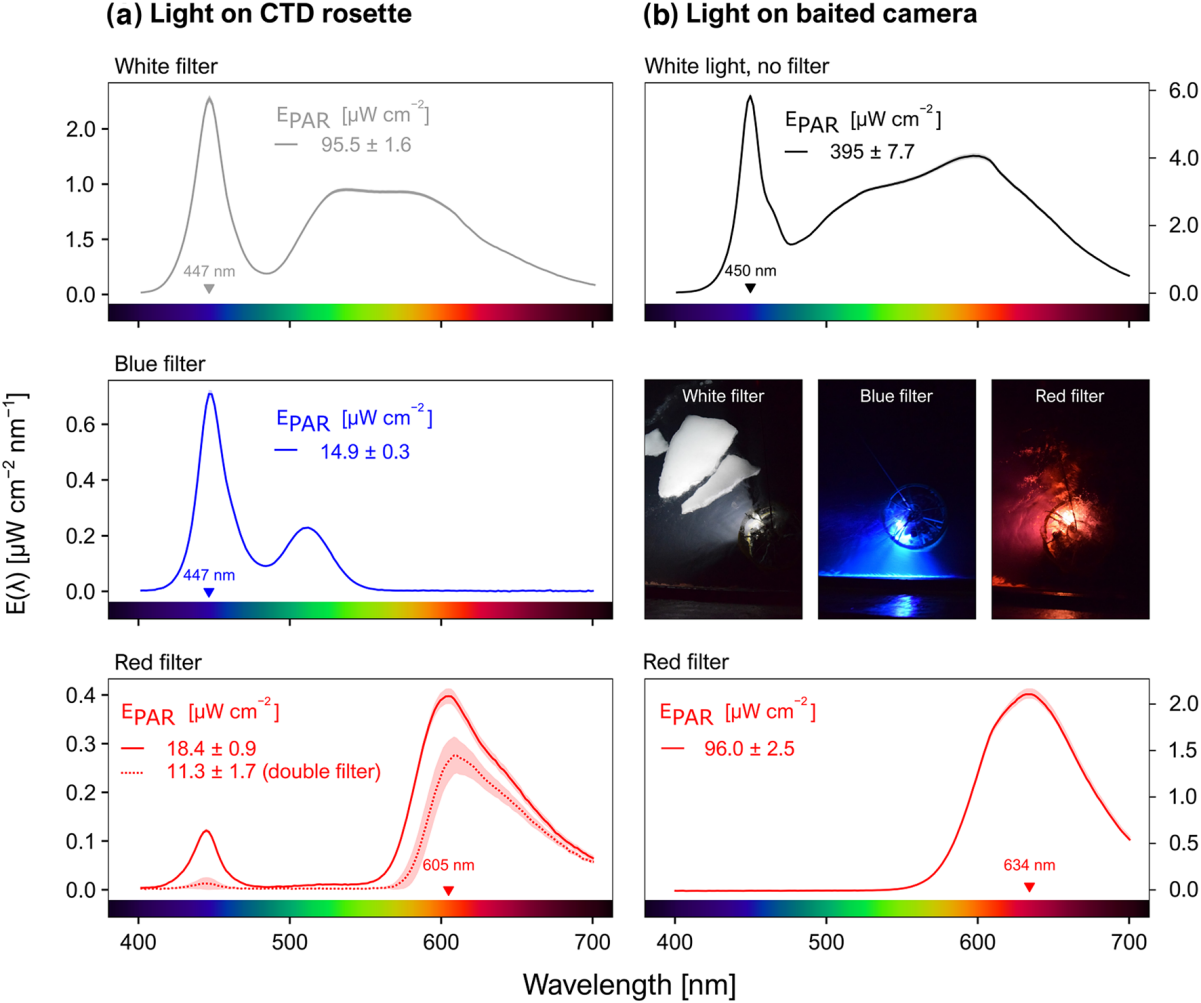
Spectral irradiance E(λ) in the range 400–700 nm, with and without different coloured filters, for the light sources mounted to (**a**) the acoustic probe and (**b**) the baited camera system. Lines show averages across triplicate measurements, with standard deviations given as envelopes. The wavelength of peak irradiance is indicated in each panel. E_PAR_ values are provided with standard deviations from triplicate measurements. Pictures of the rosette with different light colours just below the surface and taken from the *Helmer* *Hanssen* on January 2020 are included.

In Svalbard, the irradiance integrated over 400–700 nm (E_PAR_) reached 95.5 μW cm^−2^ for the white filter, 14.9 μW cm^−2^ for the blue filter, 18.4 μW cm^−2^ for a single red filter, and 11.3 μW cm^−2^ for a double red filter. Blue and red spectral irradiance peaked at 447 nm and 605 nm, respectively (Fig. 2a). Note that in all cases the spectral composition was broad, with a full width at half maximum (FWHM) of 23 nm for the blue light and 75 nm for the red light.

Analysis of absorption and backscattering spectra suggested that coloured dissolved organic matter absorption was the dominant contributor to diffuse attenuation in the blue-green wavelengths while water absorption dominated the red end of the visible spectrum. Particle backscattering signals were generally low across the entire visible spectrum. Horizontal spectral penetration distance (i.e., the range at which irradiance diminishes to 1/e of its initial value) reached ~ 8 m for blue light,  ~ 14 m for green light and ~ 6 m for the red light, indicating that the red light was attenuated stronger than light at other colours. Optical penetration depth decreases rapidly in the near infrared, where water absorption increases, but even at longer red wavelengths (e.g., 676 nm) the penetration distance was ~ 2 m in these waters (Supplementary Fig. S1 online). By applying these absorption coefficients to the distance of avoidance of each Svalbard deployment, we calculated that 87.96–99.99% of the white light, > 99% of the blue light and > 99% of the red light was attenuated at the threshold distance boundaries. Hence, the pelagic organisms detected here reacted to white light with irradiance levels < 2.62E−03 μW cm^−2^, blue light with irradiance < 3.90E−04 μW cm^−2^, and red light with irradiance < 1.43E−06 μW cm^−2^ (Supplementary Table S3 online).

For the baited camera used in Newfoundland, E_PAR_ reached 395 μW cm^−2^ for the light source without any filter. Adding the red filter reduced the E_PAR_ to 96 μW cm^−2^. White and red light peaked at 450 nm and 634 nm for a FWHM of 20 nm and 84 nm, respectively (Fig. 2b).

### Light avoidance

**Figure 3:**
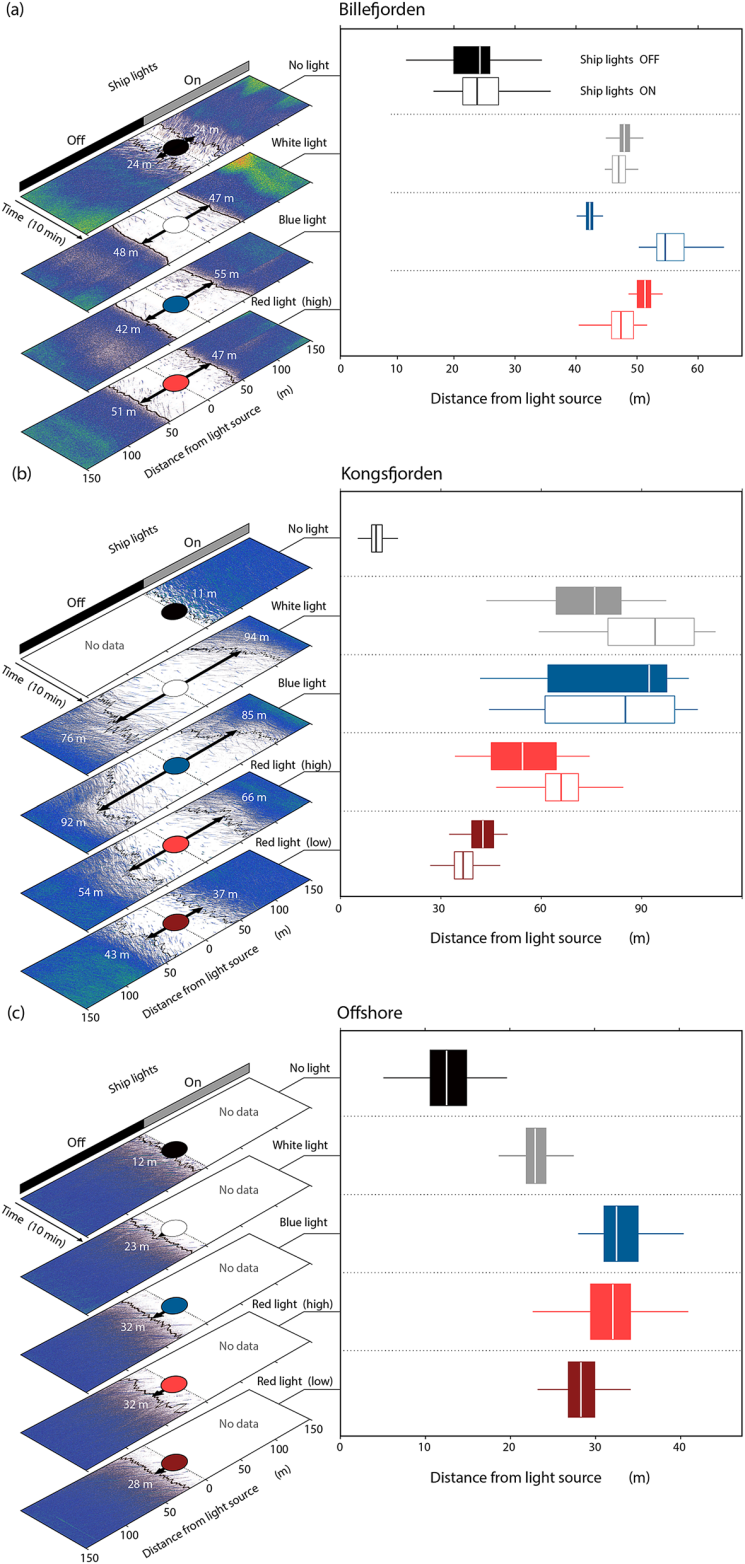
Left panels: Volume backscattering strength echograms (S_v_) from the WBAT for 10-min deployments in (**a**) Billefjorden, (**b**) Kongsfjorden, and (**c**) Offshore Svalbard under different light filters and with the ship's lights turned on or off. The black line indicates the range from the transducer to the − 90 dB (Billefjorden), − 95 dB (Kongsfjorden) or − 110 dB (offshore) backscatter threshold and the arrow indicates the median range (i.e., median avoidance distance). Right panels: Corresponding box plots of the avoidance distance. Range boxes show the 25th, 50th, and 75th and whiskers the 5th and 95th percentile, outliers are excluded.

In Svalbard, pelagic organisms were farther from the transducers of the acoustic probe when the probe’s lights were turned on (Fig. 3). The avoidance distance was significantly higher for all colours tested compared to no light (Table 1, model 2), but the light effect was weaker for the low irradiance red light compared to other probe light trials (Table 1, model 2). Within a given site and for the same probe light colours, the distance from the acoustic probe was not statistically different when the ship's lights were turned on or off, as indicated by the first generalized linear mixed model (Table 1).

Avoidance distances also varied significantly among sites (Table 1). In Billefjorden, pelagic organisms avoided the first 24 m when the lights on the acoustic probe were turned off. The avoidance distance doubled to 47–48 m when exposed to white light, 42–55 m with blue light, and 47–51 m with red lights of high irradiance (Fig. 3a). Reactions to probe light trials were even stronger in Kongsfjorden, where fish and zooplankton avoided the first 11 m when the lights on the acoustic probe were turned off, the first 76–94 m when exposed to white light, 85–92 m with blue light, 54–66 m with red light of high irradiance (18.4 μW cm^−2^) and 37–43 m with red lights of low irradiance (11.3 μW cm^−2^) (Fig. 3b).

Despite lower acoustic density, pelagic organisms also actively avoided the acoustic probe with lights on at the offshore station (Fig. 3c). With the detection threshold we used, offshore pelagic organisms avoided the first 12 m when the lights on the acoustic probe were turned off. The avoidance distance doubled to 23 m when exposed to white light, 32 m with blue light, 32 m with red light of high irradiance, and 28 m with red light of low irradiance (Fig. 3c).

**Figure 4:**
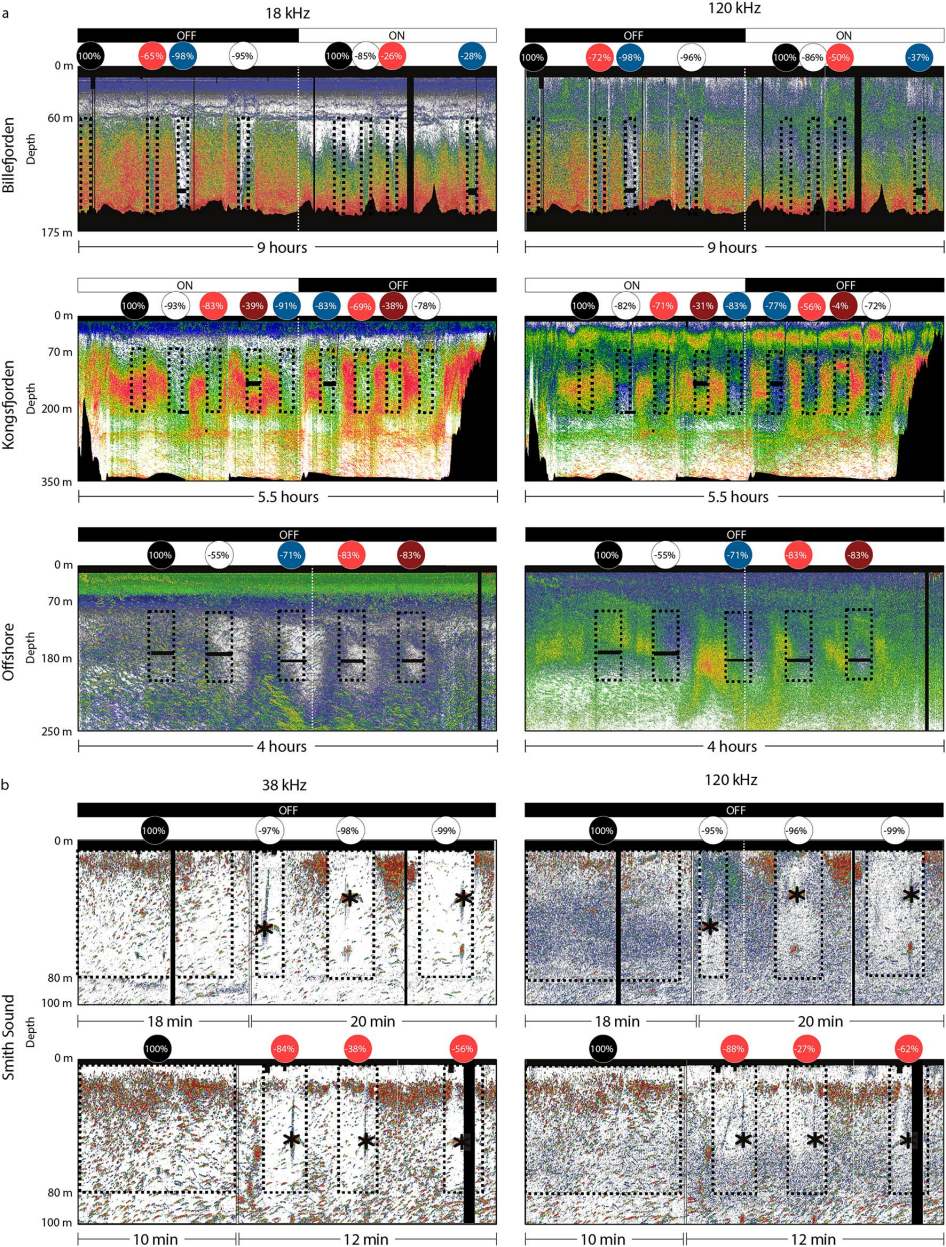
Volume backscattering strength echograms (S_v_) from the EK60 echosounder during (**a**) the Svalbard and (**b**) Newfoundland experiments. The dashed rectangles represent each deployment, and the first rectangle of each echogram represents the control deployment. The corresponding circles on top are coloured according to the filter being used and indicate the percentage reduction in area backscattering coefficient (s_a_). Black and white bars on top of the echograms indicate when the ship's lights were on and off. Black areas represent areas removed from the analyses because of noise or depths below the seafloor. The asterisks indicate the location of the baited camera during the Newfoundland experiment. Note that the signal from the camera was removed from the analyses. Higher S_v_ threshold is − 50 dB for all echograms and lower S_v_ threshold is − 90 dB for Billefjorden and Newfoundland, − 95 dB for Kongsfjorden, and − 110 dB for the offshore echograms.

The area backscattering coefficient (s_a_) was consistently lower when the lights on the probe were turned on. In Svalbard, the variation in s_a_ measured from the hull-mounted echosounder dropped by 72–96% when the white light was on, 37–98% when the blue light was on, 26–83% with the high irradiance in red light, and 4–39% with the low red irradiance (Fig. 4a). Part of the variation can be explained by the angle of the CTD cable, which sometimes drifted outside the main acoustic beam of the EK60, and by the drift of the vessel (Supplementary Fig. S2 online). In general, the reduction in s_a_ was similar at 18 kHz and 120 kHz. The reduction in acoustic backscatter was lower when the ship's lights were on because part of the community had already avoided the ensonified area in reaction to the ship's lights. The lower reduction in backscatter with the red light, especially at low irradiance, was related to a lower footprint of the light beam because of higher absorption at this wavelength, rather than representing more organisms remaining close to the light source. Indeed, when only considering 20 m around the light source the reduction in s_a_ was > 90% for all wavelengths and irradiance levels.

For the Newfoundland survey, the reduction in s_a_ varied between 95–99% with white light and 27–88% with red light (Fig. 4b). Similar to the Svalbard experiment, the lower reduction in backscatter when using the red filter was related to lower energy transmitted through the filter which resulted in a lower footprint of the light beam, rather than representing more organisms remaining close to the light source. Indeed, the reduction in backscatter was > 90% for the red light when only considering 10 m around the light source.

### Camera footage

The pelagic baited camera footage from the Newfoundland survey captured copepods and gelatinous plankton (mostly ctenophores). No fish were observed within the range of both light colours, but a squid was detected on the white light footage. The number of organism detections and rate of detections were higher with the white light than with the red light (Table 2). Similarly to what was observed on the acoustics, the lower number and rate of organism detections with the red light were related to the lower range of the light beam, which reduced the camera’s field of view and hence the number of detections.

**Table 2:** Number of organisms detected by the baited camera equipped with a white or red light during the Newfoundland survey. Note that swarms of copepods were also detected but individual copepods could not be counted.

Organisms	White light		Red light	
	40.0 min duration		36.7 min duration	
	Total number of detections	Detections per minute	Total number of detections	Detections per minute
Ctenophore	251	6.83	67	1.82
Gelatinous zooplankton	22	0.60	1	0.03
Squid	1	0.03	0	0.00
Fish	0	0.00	0	0.00
Total	274	7.46	68	1.85

## Discussion

Pelagic organisms clearly avoided both the acoustic probe and the baited camera when the instrument's lights were turned on, which resulted in an up to 99% diminution of backscatter. Pelagic organisms that can be detected at 18 kHz, 38 kHz and 120 kHz include fish, meso- and macrozooplankton. While the species composition in the survey areas was not assessed during this survey, decades of sampling in Svalbard waters demonstrated that polar cod (*Boreogadus saida*), Atlantic cod, capelin (*Mallotus villosus*) and/or juvenile redfish (*Sebastes* spp.) dominate the pelagic fish community^33,36,47^. Zooplankton scatterers that could be detected by the WBAT at 38 kHz in Svalbard comprise krill, amphipods (*Themisto* spp.), pteropods, siphonophores, swarms of copepods and chaetognaths^35,48^, and large jellyfish^49^. Krill are usually attracted to light^4^, and the clear avoidance pattern observed here thus suggests that they were not abundant. In contrast, copepods are highly abundant in Svalbard fjords, where they are known to avoid artificial light^3^. Some jellyfish also avoid artificial light^50^. The reaction of *Themisto* and pteropods to artificial light is not documented. While it is impossible to know exactly which species were avoiding the light source, the strong reduction in backscatter for all experiments and at all frequencies suggests that the use of artificial light on scientific instruments does not capture the real state of these high Arctic ecosystems.

Zooplankton groups occurring in Svalbard are also present in boreal regions. The reaction to artificial light mounted on instruments is thus most likely not limited to the high Arctic during the polar night, but also applies to other ecosystems at lower latitudes. This reasoning is supported by the avoidance of the light from the baited camera deployed in Newfoundland. Similarly to the Svalbard area, copepods, amphipods and krill are the main zooplankton taxa in Newfoundland^51^, while herring, capelin and Atlantic cod are the main pelagic fish species. Although only zooplankton was observed on the pelagic baited camera, we also observed a strong avoidance behaviour of the same baited camera by capelin when deployed on the seabed using white light during a similar survey conducted in western Newfoundland (Supplementary Fig. S3 online). It is, however, important to note that positive or negative phototactic responses can vary from one species to the other^5^. Moreover, it is possible that species avoiding diffuse light, as used here, are attracted to more directional light sources^10,52^. More studies in different regions and ecosystems are needed to better quantify the reaction to artificial light from different fish and zooplankton species.

Shadowing, turbulence and low-frequency sound created when lowering and retrieving the probe, or when it moves, can be detected by pelagic organisms and contribute to their avoidance of probes^53–55^. These factors likely explain the 12–24 m avoidance from the probe measured when the probe’s lights were off, which is in accordance with previous observations (e.g., 7–20 m for small organisms such as zooplankton^53^). However, our results suggest that adding light on the instrument more than doubles the avoidance distance compared to that from the probe itself (Fig. 3).

Pelagic organisms reacted to all wavelengths (colours) tested. It is possible that pelagic organisms reacted to the blue-green peak of lower irradiance when only one red filter was used in Svalbard (Fig. 2). However, this peak almost completely disappeared with two filters and was absent from the light used with the baited camera (Fig. 2a,b), both of which resulted in a spectrum mainly confined to wavelengths between 575 and > 700 nm. The avoidance response to these light sources confirmed that pelagic organisms reacted to red light. However, these red lights covered a broad spectrum extending from the higher end of the green wavelengths and it is possible that pelagic organisms would not have responded to far red light with a narrow bandwidth (e.g., > 650 nm). For example, Raymond and Widder^56^ reported a minimal response to longer wavelengths of red light (680 nm) compared to white light from deep-sea fishes. Using infrared light could be another option because these wavelengths are unobtrusive to most animals. Unfortunately, the downside of longer wavelengths is their very limited range, for example of ~ 1 m for infrared (^32^ and references therein^57^). Widder et al.^28^ suggested using red light instead of white light on optical instruments to mitigate avoidance biases during optical surveys. Our observations rather suggest that using visible red light with a broad spectral composition in the 575–700 nm wavelengths to survey pelagic organisms does not prevent phototactic behaviours, at least within the epipelagic realm. Similarly to our observations, Marchesan et al.^5^ reported reactions over the whole colour spectrum, from violet to red, in some epipelagic fishes, notably the gilthead seabream *Sparus auratus*.

We clearly showed that pelagic organisms avoided all wavelengths, but this study was not designed to test the effect of intensity as only red light was tested at varying irradiance levels. To better evaluate the respective effects of spectrum and irradiance levels, future studies could use spectra-specific LED lights with similar dimming capacities to test the distance of avoidance at varying intensities but consistent wavelengths. Reducing the red irradiance (E_red_) by 37%, from 18.4 to 11.3 μW cm^−2^, diminished the average distance of avoidance from 54–66 m to 37–43 m in Kongsfjorden and from 32 to 28 m offshore (Fig. 3). Yet, the avoidance remained significantly higher than when the probe's lights were off. Similarly, Trenkel et al.^27^ and Marchesan et al.^5^ observed stronger avoidance behaviour from fish at higher irradiance levels. However, comparing the distance of avoidance for all irradiance levels used in Svalbard suggests that increasing the irradiance level increased the distance of avoidance until a certain intensity (ca. 20 μW cm^−2^), but that the median distance of avoidance did not vary significantly passed that threshold (Supplementary Fig. S4 online). This could explain why Peña et al.^12^ did not report changes in avoidance behaviour when progressively dimming their white light. The maximum distance of avoidance reached 94 m in Kongsfjorden, 55 m in Billefjorden, and only 32 m offshore. Surprisingly, the distance of avoidance from white light at the offshore location was shorter compared to other colours, while it was the opposite in Kongsfjorden and Billefjorden. The exact reason behind these differences remains unknown but could be related to different assemblages of zooplankton and fish. Yet, in all cases the response was triggered by switching the light on and was of similar magnitude irrespective of the wavelength of the light, with red light having only marginally different impacts than the other lights.

Our results from Svalbard reveal the potential for context-specific behaviours to interact with light conditions. Using similar experimental set ups, Peña et al*.*^12^ observed avoidance by lantern fish of white LED lights dimmed from 3800 millivolts, but not of red light (660 nm with a 4200 millivolts dimming). Similarly, Underwood et al*.*^11^ observed mesopelagic fish avoidance of white (442 or 546 nm), blue (462 nm), and green light (516 nm) with intensities between 123–2200 μW cm^−2^, but not of red light (633 nm) with intensity of 90 μW cm^−2^. Here, in contrast, we measured avoidance of at least 23 m and a significant reduction in backscatter from all light wavelengths, including red. The discrepancy between our observations and that of Peña et al*.*^12^ and Underwood et al.^11^ might originate from different pelagic communities. Both of these studies conducted their experiments at mesopelagic depths (> 200 m), in deep oceanic basins, and measured the avoidance response of mesopelagic fish. Red light is absorbed more rapidly than other colours in seawater and ambient surface irradiance will attenuate to insignificant levels within the top ca. 50 m. Most deep-water and mesopelagic fish, including lanternfish, thus evolved less chromatic (colour) sensitivity, with peak detection at shorter wavelengths centered around blue to green wavelengths (380 nm to 620 nm) and less so to longer red wavelengths^58–60^, even though some species can be sensitive to red light at short ranges^59^. Here, however, we deployed our acoustic probe at 125–140 m and the baited camera at 35–50 m and targeted epipelagic species. These species could be exposed to, and more likely to detect, shorter-wavelength red light from the sun.

Molecular studies on colour vision of certain fish present in the study areas suggest the expression of diverse blue- and green-sensitive opsins^61^, the proteins which tune colour vision in retinal cone cells, along with the evolutionary loss of both short (ultraviolet) and long (red) wavelength opsins. But even a green-sensitive cone can still confer some long-wavelength sensitivity at sufficient intensities. This is likely the case for Atlantic cod, the fish species in our sampling areas with the best studied visual system. Anthony and Hawkins^62^ reported fairly broad visual sensitivity for Atlantic cod in behavioral and electrophysiological studies, with spectral sensitivity maxima in the blue and green (490 and 550 nm) and some evidence of long-wavelength sensitivity under light adaptation. Since the spectral absorbance of rhodopsin visual pigments is ~ 100 nm FWHM^63^, it is reasonable to assume that green/yellow sensitive visual pigments in fish could detect red light, particularly the red lights used in our study given their relatively broad spectral composition despite having spectral maxima above 600 nm. Among the taxa in our study areas, fish are more likely to have broader spectral sensitivities than the marine invertebrate zooplankters as most of the latter are monochromatic with spectral sensitivities in the blue-green (e.g.^64–66^). However, invertebrates could also detect longer-wavelength light with a blue/green sensitive visual pigment as described above for fish if the light is sufficiently bright^67^.

Each of our Svalbard measurements was limited to 10 min (Fig. 4a), and it is possible that fish and zooplankton would have become acclimatized to the light field after a longer period of time and would have returned to reoccupy the sampling volume. We conducted an experiment with artificial light from a research vessel in the same region and observed a partial recovery towards "normal" distribution 2.5 h after the lights were turned on (unpublished data). Yet, optical probes are generally lowered and retrieved at speeds between 0.3–1 m s^−1^, which means that the same volume of water is only sampled for a few seconds. Given the fast response to light observed here, in the order of a few seconds, our results strongly suggest that any of these instruments deployed as a probe to monitor zooplankton and/or fish would be biased by avoidance behaviours, especially by relatively large and motile organisms. It is, however, not clear if strobe lights would result in similar phototactic responses (e.g.^32^).

Our pelagic trials in Newfoundland were shorter (≤ 40 min; Table 2) than usual baited camera deployments, which generally last several hours (e.g.^26^). It is possible that pelagic organisms come back into the sampling view after some time, being attracted by the bait or by their prey in the light beam. Intraspecific differences could also complicate the analyses, with some individuals being attracted to the bait with others preferring to remain outside of the light field. At the moment, however, it is impossible to know if all animals get accustomed after a few hours or if some individuals or species continue to avoid, or to be attracted to, light throughout the duration of longer deployments. Including a control deployment of the baited camera with lights off to assess the avoidance of the instrument itself would have also increased the interpretability of these measurements. What was clear from the combined acoustic and baited camera data, however, was that (1) pelagic organisms avoided the baited camera with both white and red lights; and (2) despite white light trials exhibiting consistently lower reductions in area backscattering (s_a_) than within red light trials (Fig. 4b), white light resulted in higher pelagic species abundances and rates of occurrence in the camera footage (Table 2). These seemingly contradictory results between acoustic (white light = lower abundances) and optical measures (white light = higher abundances) illustrate both the apparent influence of light attenuation (white < red) on optical detections and the utility of multi-method experiments to quantify such biases^14^.

## Conclusion

Despite an increased interest in using cameras for biodiversity monitoring and fish stock assessments^14,68^, we conclude that presence/absence observations and rates of detections from optical instruments with visible light probes should be interpreted carefully. While several questions remain in terms of phototactic reaction from different species to artificial light of varying characteristics (e.g. diffuse vs. directional), irradiance levels, colour composition, and exposition periods, we demonstrate a clear avoidance response by pelagic organisms to several wavelengths, including visible red, at both high and mid-latitudes. Reliable biodiversity, density and abundance estimates will not be achieved without further studies to elucidate the behavioural responses to illuminated optical instruments.

## Supplementary Information

Supplementary Information.

## Data Availability

The datasets generated during the current study are available from the corresponding author on reasonable request.
