# Supplementary material for: Pelagic organisms avoid white, blue, and red artificial light from scientific instruments
Source: Sci Rep. 2021 Jul 22;11:14941. doi: 10.1038/s41598-021-94355-6 (PMC8298562; doi:10.1038/s41598-021-94355-6)
Supplement: Supplementary file 1 — Supplementary Information. [file 41598_2021_94355_MOESM1_ESM.pdf]

Pelagic organisms avoid white, blue, and red artificial light from scientific instruments

Maxime Geoffroy[1,2]*, Tom Langbehn[3], Pierre Priou[1], Øystein Varpe[3,4], Geir Johnsen[5,6], Arnault LeBris[1], Jonathan A. D. Fisher[1], Malin Daase[2], David McKee[7,2], Jonathan Cohen[8], Jørgen Berge[2,5,6]

[1]Centre for Fisheries Ecosystems Research, Fisheries and Marine Institute of Memorial University of Newfoundland, St. John's, NL, Canada

[2]Department of Arctic and Marine Biology, The Arctic University of Norway, Tromsø, Norway

[3]Department of Biological Sciences, University of Bergen, Bergen 5020, Norway

[4] Norwegian Institute for Nature Research, 5006 Bergen, Norway

[5]Centre for Autonomous Marine Operations and Systems, Department of Biology, Norwegian University of Science and Technology, Trondheim, Norway

[6]University Centre in Svalbard, Longyearbyen, Norway

[7]Physics Department, University of Strathclyde, Scotland

[8]School of Marine Science and Policy, University of Delaware, Lewes, USA

*Corresponding author (maxime.geoffroy@mi.mun.ca)

Supplementary materials

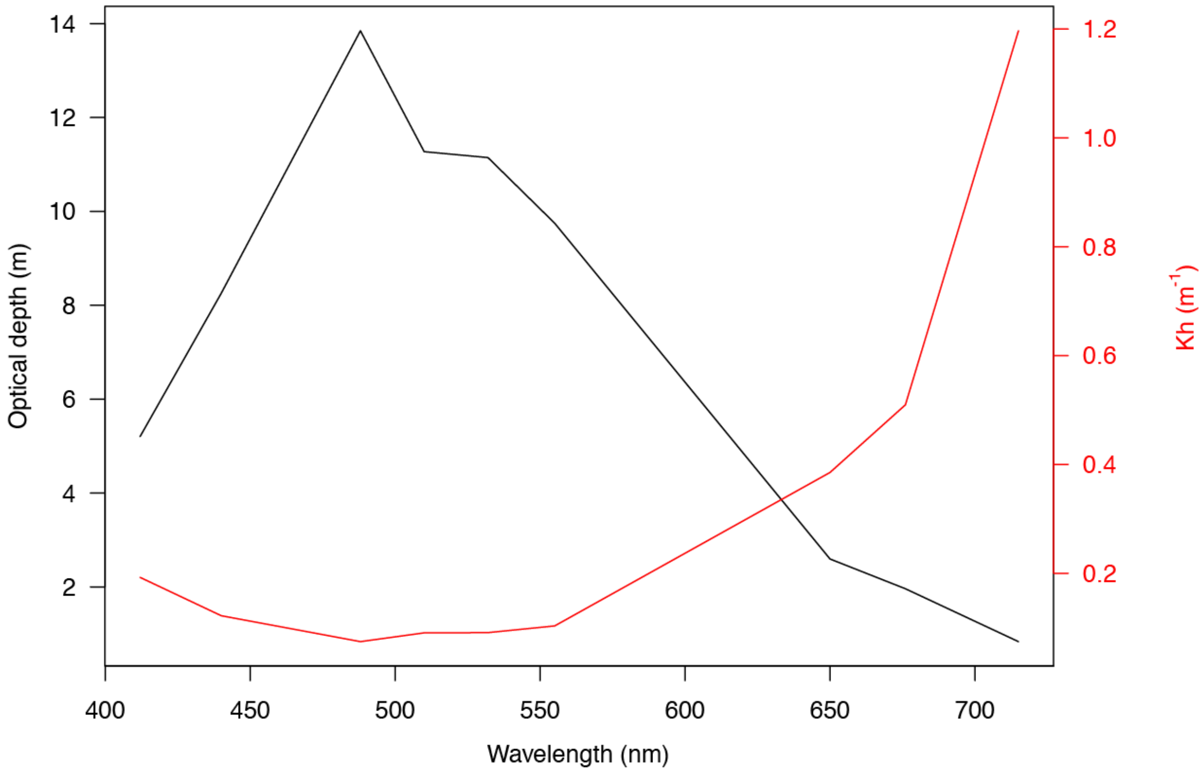

Supplementary Figure S1. Average optical depth (m) and diffuse attenuation coefficient (K_h) within the range 400-700 nm in Svalbard waters.

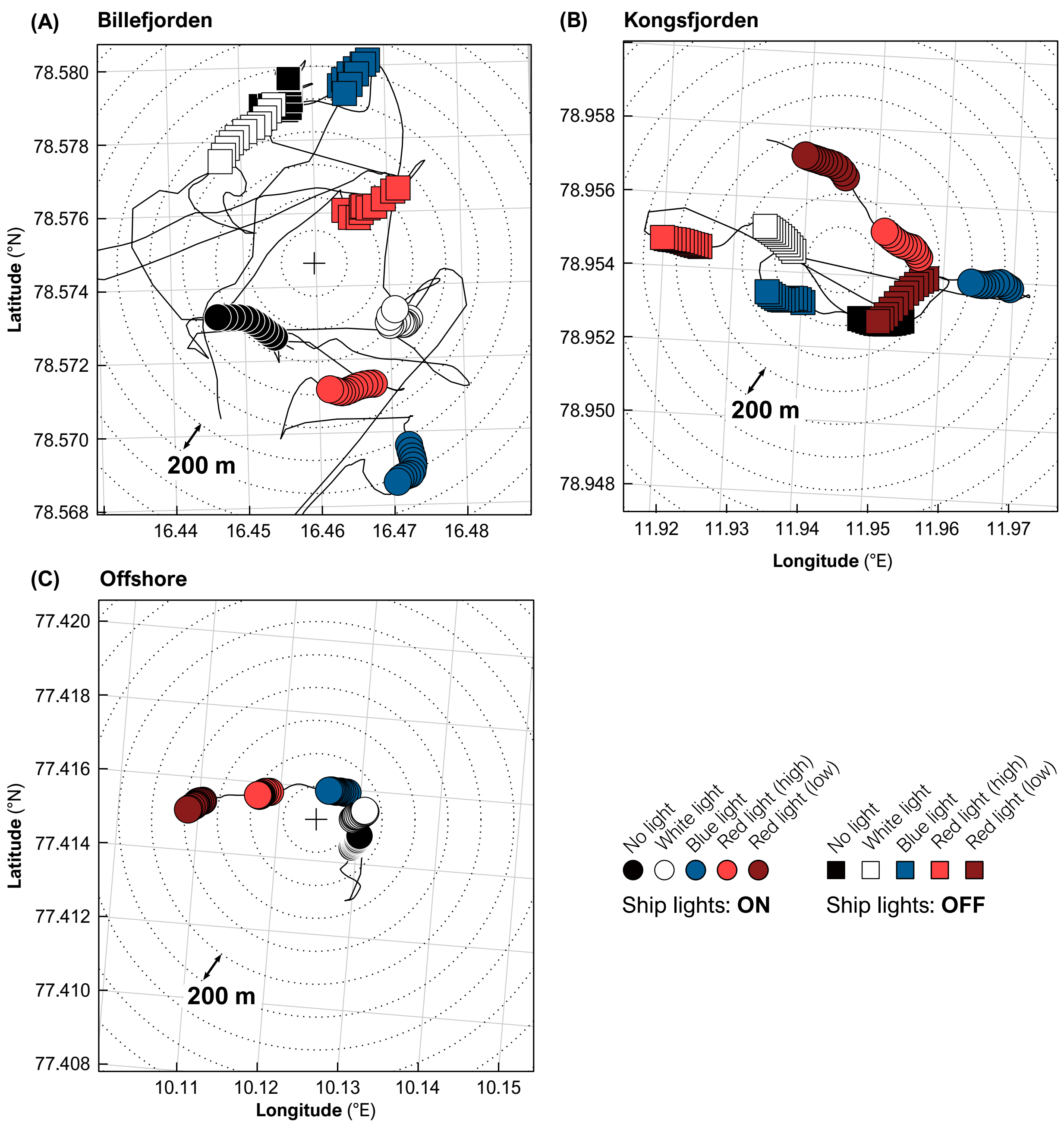

Supplementary Figure S2. Drifting trajectory of the R/V *Helmer Hanssen* during the experiments in Billefjorden, Kongsfjorden, and offshore Svalbard.

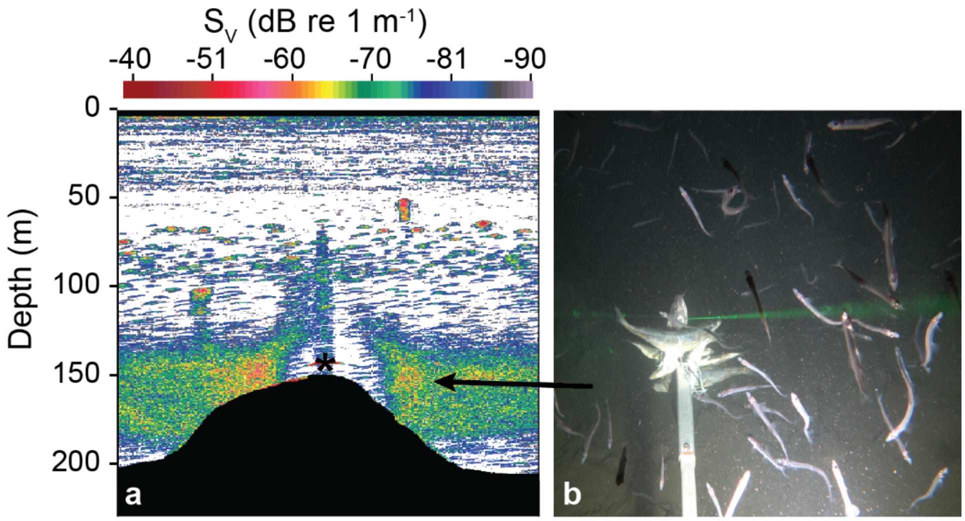

Supplementary Figure S3. (a) S_v echograms from the EK60 at 38 kHz during a survey in Bonne Bay on the west coast of Newfoundland in October 2018. Black areas represent areas removed from the analyses because of noise or depths below the seafloor and the asterisk indicates the location of the baited camera equipped with a white LED light. (b) Snapshot of capelin from the baited camera footage from within the scattering layer found close to the seafloor.

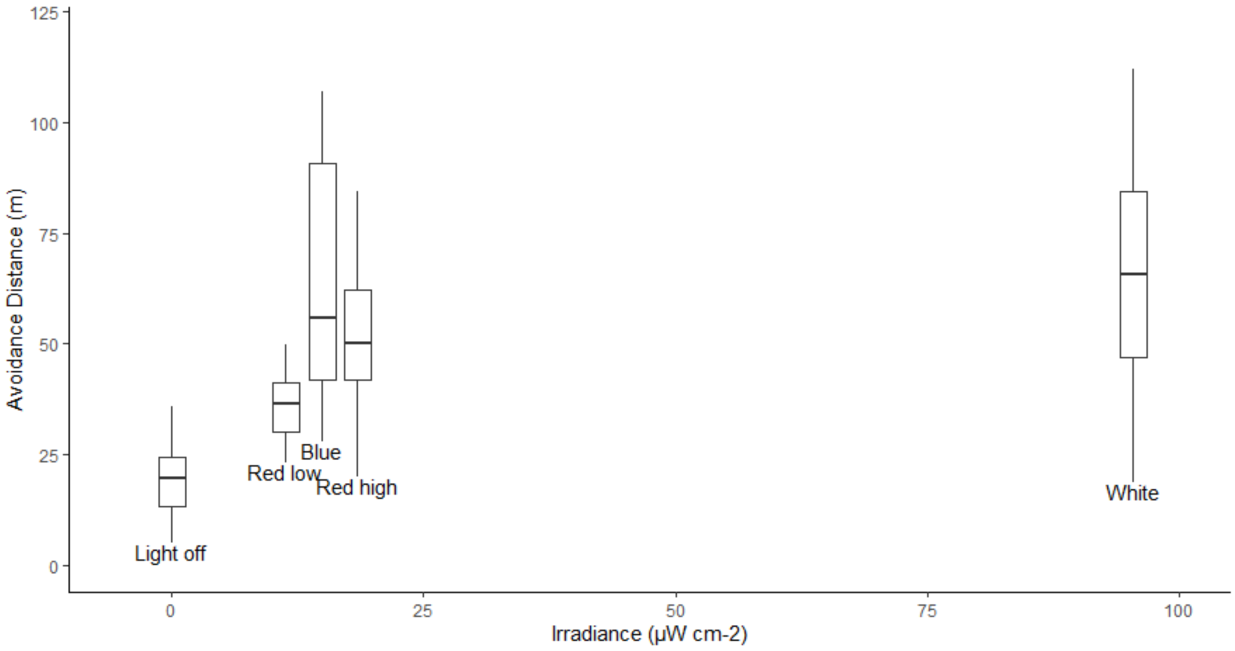

Supplementary Figure S4. Boxplot of the median distance of avoidance relative to the irradiance levels for all experiments conducted in Svalbard. Range boxes show the 25th, 50th, and 75th and whiskers the 5th and 95th percentile, outliers are excluded.

Supplementary Table S1. Settings of the EK60 echosounder during the Svalbard and Newfoundland experiments.

	Svalbard		Newfoundland	
**Frequency (kHz)**	18	120	38	120
**Power (W)**	2000	250	2000	500
**Pulse length (ms)**	1.024	1.024	1.024	1.024
**Ping rate (ping sec^{-1})**	1	1	1	1
**Beam width (°)**	10	7	7	7

Supplementary Table S2. Settings of the Best Bottom Candidate algorithm in Echoview used to measure the avoidance distance. A scattering threshold of -90 dB was used in Billefjorden, -95 dB in Kongsfjorden, and a -110 dB threshold was applied offshore Svalbard. Avoidance distance was smoothed over 15 pings before being averaged.

**Maximum dropout (samples)**	50
**Window radius (samples)**	8
**Minimum peak asymmetry**	-0.50

Supplementary Table S3. Percentage of the probe light and irradiance level at mean distance of avoidance compared to irradiance level at 1 m from the source for each deployment in Svalbard.

**Region**	**Ship's lights**	**Colour**	**Irradiance level at 1 m from the source at peak wavelength (μW cm^{-2})**	**% of probe light at distance of avoidance**	**Irradiance level at distance of avoidance**
Billefjorden	OFF	White	95.5	4.01E-01	3.83E-01
Billefjorden	ON	White	95.5	4.50E-01	4.29E-01
Billefjorden	OFF	Blue	14.9	6.12E-01	9.12E-02
Billefjorden	ON	Blue	14.9	1.12E-01	1.67E-02
Billefjorden	OFF	Red high	18.4	4.24E-04	7.79E-05
Billefjorden	ON	Red high	18.4	1.12E-03	2.06E-04
Kongsfjorden	OFF	White	95.5	1.74E-02	1.66E-02
Kongsfjorden	ON	White	95.5	2.74E-03	2.62E-03
Kongsfjorden	OFF	Blue	14.9	2.62E-03	3.90E-04
Kongsfjorden	ON	Blue	14.9	3.37E-03	5.03E-04
Kongsfjorden	OFF	Red high	18.4	1.53E-04	2.81E-05
Kongsfjorden	ON	Red high	18.4	7.80E-06	1.43E-06
Kongsfjorden	OFF	Red low	11.3	3.00E-03	3.39E-04
Kongsfjorden	ON	Red low	11.3	1.04E-02	1.17E-03
Offshore	OFF	White	95.5	1.20E+01	1.15E+01
Offshore	OFF	Blue	14.9	3.58E+00	5.34E-01
Offshore	OFF	Red high	18.4	7.68E-02	1.41E-02
Offshore	OFF	Red low	11.3	1.54E-01	1.74E-02
